# Verrucisidinol and Verrucosidinol Acetate, Two Pyrone-Type Polyketides Isolated from a Marine Derived Fungus, *Penicillium aurantiogriseum*

**DOI:** 10.3390/md8112744

**Published:** 2010-11-01

**Authors:** Ke Yu, Biao Ren, Junli Wei, Caixia Chen, Jinsheng Sun, Fuhang Song, Huanqin Dai, Lixin Zhang

**Affiliations:** 1 Chinese Academy of Sciences Key Laboratory of Pathogenic Microbiology and Immunology, Institute of Microbiology, Chinese Academy of Sciences, Beijing 100190, China; 2 Graduate University of Chinese Academy of Sciences, Beijing 100190, China; 3 Tianjin Normal University, Tianjin 300387, China

**Keywords:** marine fungus, *Penicillium aurantiogriseum*, verrucosidin

## Abstract

The new secondary metabolites verrucosidinol (**1**) and its derivative verrucosidinol acetate (**2**), together with a potent neurotoxin verrucosidin (**3**), a congener norverrucosidin (**4**) and a mixture of two known phytotoxic metabolites terrestric acids (**5** and **6**), were isolated from the marine derived fungus *Penicillium aurantiogriseum*. Verrucosidinol has a ring-opened ethylene oxide moiety in the polyene α-pyrone skeleton, and verrucosidinol acetate is its acetate derivative. The chemical structures were determined by comparing with literature data and a combination of spectroscopic techniques, including high resolution mass spectrum and two-dimentional nuclear magnetic resonance spectroscopic analysis.

## 1. Introduction

Compared with the terrestrial environment, the marine habitat has some unique characteristics, such as high-pressure, high-salt, oxygen deficiency, and low nutrition. To survive in this harsh environment, oceanic life forms have evolved specific physiological and biochemical pathways to produce secondary metabolites [1,2]. These chemicals may help them to guard against predators, prevent the adhesion of marine algae, and play an important part in the complicated signal transduction between different species. Among them, marine microorganisms in particular have the wide range of adaptability, leading to the distinctive ability to produce unprecedented compounds which have novel structures and significant bioactivity [3]. Natural products discovery from marine microbes has enjoyed a renaissance in recent years [4]. Many lead compounds have been obtained from marine microorganisms, such as salinosporamide A, azamerone, mechercharmycins A, and marinomycin A, thereby demonstrating that the marine environment, with its diverse microflora, is a very promising resource [4]. In our search for bioactive compounds from the marine environment, a marine microbial extract library was constructed and screened for various biological activities. The antifungal potentiator beauvericin has already been identified from this particular library, demonstrating that potentially more bioactive compounds could be found and some of the bioactive metabolites could be genetically engineered to further improve the yields [5–9].

**Figure 1:**
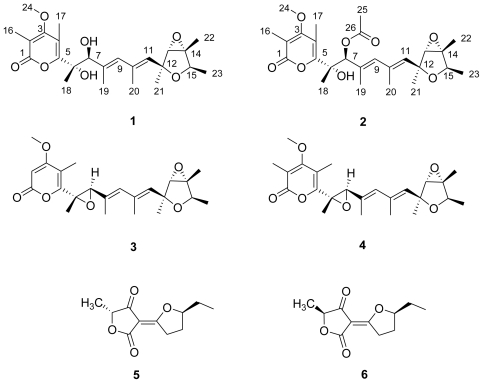
Six (**1**–**6**) compounds isolated from the extract of a fungus *Penicillium verrucosum*.

*Penicillium* is an important genus in toxigenic fungi. Chemical investigation of *Penicillium verrucosum* has afforded an immunotoxin ochratoxin A, which threatens human health through contaminated food in a wide region [10]. An outbreak of neurological disease in cattle has been reported in the USA [11]. The mycotoxin verrucosidin, from another variety *Penicillium verrucosum* var. *cyclopium*, was subsequently found to be responsible for the outbreak [12]. Verrucosidin is a down-regulator of UPR (unfolded protein response) induced genes, such as *grp*78, resulting in selective cell death under strict hypoglycemic conditions [13]. Furthermore, the nephrotoxic product from an adjacent species *Penicillium aurantiogriseum* is a possible factor in Balkan endemic nephropathy which has been confirmed in rats and hamsters [14,15]. During the chemical study of this genus, some cytotoxic quinazoline alkaloids, and an antitumor compound, anicequol, have been identified [16,17]. The chemical diversity of this genus and the peculiarity of the marine biotope prompted us to investigate the extract from a strain of *Penicillium aurantiogriseum*. With the aid of HPLC-diode array analysis, two new verrucosidin analogs, named verrucosidinol (**1**) and verrucosidinol acetate (**2**), together with norverrucosidin (**3**) and verrucosidin (**4**) were isolated from this marine-derived fungus (Figure 1). During the extraction and isolation procedure, a mixture of the phytotoxic metabolites terrestric acids (**5** and **6**) was also obtained [18]. Herein, we report the isolation, structure elucidation, and bioactivities of these compounds.

## 2. Results and Discussion

Compound **1** was obtained as yellow oil. The HRESIMS, in combination with ^1^H and ^13^C NMR data, indicated a composition of C_24_H_34_O_7_, requiring 8 unsaturations. The NMR spectra of **1** were compared to that of verrucosidin and found to be almost the same except for H-6/C-6, H-7/C-7, C-8 and C-10 [19–22]. Taking into account the degree of unsaturation, the molecule should have a ring-opened structure. The ^1^H and ^13^C NMR spectra of compound **1** exhibited signals for 32 protons and 24 distinct carbons, respectively. A heteronuclear multiple-quantum coherency (HMQC) NMR experiment established all one-bond ^1^H-^13^C connections as indicated in Table 1. The heteronuclear multiplebond correlation (HMBC) NMR spectrum displayed ^1^H-^13^C couplings from the methyl protons 18-H (δ_H_ 1.40) to C-5 (δ_C_ 159.7), C-6 (δ_C_ 78.8) and C-7 (δ_C_ 79.8), from the methyl protons 19-H (δ_H_ 1.82) to C-7 (δ_C_ 79.8), C-8 (δ_C_ 133.9) and C-9 (δ_C_ 134.3), and from the methyl protons 20-H (δ_H_ 1.89) to C-9 (δ_C_ 134.3), C-10 (δ_C_ 134.4), and C-11 (δ_C_ 133.0), thereby establishing the existence of a heptadiene moiety. The ^1^H-^1^H COSY NMR experiment revealed correlations from 9-H to 19-H, 9-H to 20-H and 20-H to 11-H supporting this conclusion. The ^13^C NMR chemical shifts of five downfield signals corresponding to C-1 (δ_C_ 165.0), C-2 (δ_C_ 110.4), C-3 (δ_C_ 169.2), C-4 (δ_C_ 111.8) and C-5 (δ_C_ 159.7), were in close agreement to those for an *α*-pyrone unit [19,22,23]. Two singlet olefinic methyl protons 16-H (δ_H_ 2.01) and 17-H (δ_H_ 2.21) exhibited HMBC correlations to their adjacent carbons in the *α*-pyrone moiety, C-1 (δ_C_ 165.0), C-2 (δ_C_ 110.4) and C-3 (δ_C_ 169.2); C-3 (δ_C_ 169.2), C-4 (δ_C_ 111.8), and C-5 (δ_C_ 159.7), respectively, as indicated in Table 1. Additionally, the methoxy protons 24-H (δ_H_ 3.79) revealed ^1^H-^13^C coupling to a quaternary carbon C-3 (δ_C_ 169.2), indicating the connection of the methoxy moiety to the C-3 position in the *α*-pyrone substructure. The long range couplings from olefinic methyl protons 17-H (δ_H_ 2.21) to C-5 (δ_C_ 159.7), from the methyl protons 18-H (δ_H_ 1.40) to C-5, and from the methine proton 7-H (δ_H_ 4.61) to C-5, indicated a connection between the partial heptadiene structure and the *α*-pyrone moiety. The quartet methine protons 15-H (δ_H_ 4.12), the doublet methyl protons 23-H(δ_H_ 1.18), and the ^1^H-^1^H COSY signal between them suggested the methyl group was located at C-15. The long range couplings from the methyl protons 22-H (δ_H_ 1.47) to C-14 (δ_C_ 67.4) and C-15 (δ_C_ 76.7), and from the doublet methyl protons 23-H (δ_H_ 1.18) to C-14 (δ_C_ 67.4) and C-15 (δ_C_ 76.7), linked the quaternary carbon C-14 to C-15. ^1^H-^13^C long range couplings from the methyl protons 21-H (δ_H_ 1.41) to C-11 (δ_C_ 133.0), C-12 (δ_C_ 80.1) and C-13 (δ_C_ 67.5), from an oxygenated methine proton 13-H (δ_H_ 3.43) to C-12 (δ_C_ 80.1), and from an olefinic methine proton 11-H (δ_H_ 5.53) to C-12 (δ_C_ 80.1) connected the quaternary carbon C-12 to the heptadiene moiety. The two oxygenated carbons C-13 (δ_C_ 67.5) and C-14 (δ_C_ 67.4) were concluded to form an epoxide ring. In contrast, two further deshielded oxygenated carbons C-6 (δ_C_ 78.8) and C-7 (δ_C_ 79.8), which had the higher chemical shifts, were connected to hydroxy groups respectively. This speculation was confirmed by the HRESIMS data. The molecular formula of 1 was determined from HRESIMS as C_24_H_34_O_7_, corresponding to the difference of one water molecule in the molecular formula between **1** and **4.** Therefore, compound **1** was suggested to be a ring-opened derivative of **4**.

On the basis of identical chemical shift values and coupling constants for both compounds, the relative configuration of the tetrahydrofuran in **1** was proposed to be the same as that of verrucosidin, which was assigned by X-ray crystal diffraction [24]. Key NOESY enhancements between 11-H and 13-H, 13-H and 22-H, 23-H and 11-H, 22-H and 23-H, as well as a weak enhancement between 15-H and 21-H supported the orientations of these groups in tetrahydrofuran ring (Figure 3). The geometry of C-8 was confirmed to be *E* by the NOE observation between 7-H and 9-H. And the NOESY enhancements between 7-H and 17-H, 18-H and 19-H, determined their steric configuration respectively.

**Figure 2:**
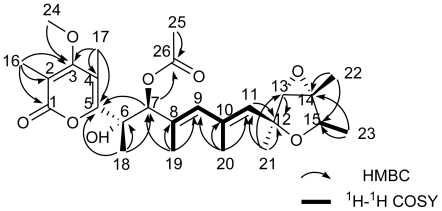
HMBC and ^1^H-^1^H COSY analysis of **2**.

**Figure 3:**
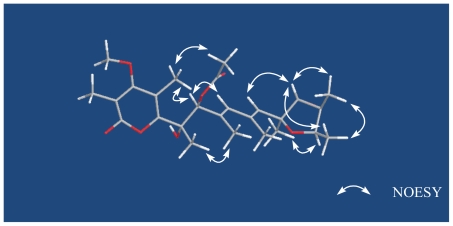
NOESY NMR analysis of **2**.

Compound **2** possessed a molecular formula of C_26_H_36_O_8_ as determined by HRESIMS and NMR data. An additional C_2_H_2_O was the only difference compared with **1**. Considering the similarity of the NMR data (Table 1), compound 2 was most probably an acetate derivative of 1. The characteristic NMR signals, quaternary carbon C-26 (δ_C_ 169.4), primary carbon C-25 (δ_C_ 21.0) and singlet methyl protons 25-H (δ_H_ 2.06), confirmed this. The acetate group was unambiguously attached to C-7 (δ_C_ 82.1), which was concluded from the correlation between H-7 (δ_H_ 5.43) and C-26 in the HMBC, shown in Figure 2. Further analysis of the HSQC, ^1^H-^1^H COSY and HMBC NMR data of 2 enabled the establishment of its structure. The NOESY NMR data of 2 was well matched to that of 1, except for an additional enhancement between 17-H and 25-H, which determined the relative configuration of the acetate ester group in position 7 (Figure 3).

Compound **4** was obtained as colorless plates and established as verrucosidin by comparison of the spectroscopic data reported in the literature [12,19,24]. Verrucosidin is a potent neurotoxin which can cause tremble and paralysis experimentally in mice [11,12]. It was first isolated from *Penicillium verrucosum* var. *cyclopium*, and then often found from the contaminated dry-cured ham [25]. Recently, the genotoxicity and chaperone down-regulate activity of verrucosidin has been reported [13].

Compound **3** was isolated as yellow oil. When its mass spectrum was compared with **4**, the lack of one methyl group was concluded. By comparison with the reported data for normethylverrucosidin, its structure was assigned [12]. Normethylverrucosidin, together with verrucosidin and terrestric acid, plays an important role in chemotaxonomy of terverticillate penicillia [26].

A mixture of compound **5** and **6** was obtained as colorless needles and determined as a mixture of terrestric acids. Since the corresponding peaks for both the *cis*- and *trans*-isomers were observed in the NMR data, it was established that this was a mixture of both *cis*- and *trans*-terrestric acids [18,27]. Terrestric acid was first isolated from *Penicillium terrestre* [28], and then from *Pyricularia oryzae* [18], and it has been reported to display phytotoxic activity.

Although acetic acid was used during the isolation, none of derivatives of normethylverrucosidin were detected, which suggested that compounds **1** and **2** should be generated by the fungus instead of being artificial ring-opened product of **4**. Considering the structural relationship between **1** and **2**, the possibility still existed that compound **2** is the acetate esterified product brought about as an artifact process of **1**.

The compounds were also tested for bioactivity against methicillin resistant *Staphylococcus aureus*, *Pseudomonas aeruginosa*, *Candida albicans* SC5314, and synergistic antifungal activity with ketoconazole. Since all minimum inhibitory concentrations (MICs) of tested compounds were ≥64 μg/mL, no significant activity against the above-mentioned microbes was concluded.

## 3. Experimental Section

### 3.1. General Experimental Procedures

Optical rotations were measured on a Perkin-Elmer 241 MC polarimeter. IR spectra were recorded on a Nicolet 5700 FT-IR microscope spectrophotometer (FT-IR Microscope Transmission). NMR spectra were recorded on a Varian Inova 500 MHz spectrophotometer at 500.103 MHz for ^1^H and 125.762 MHz for ^13^C in CDCl_3_ using solvent signals (CHCl_3_; δ_H_ 7.26/δ_C_ 77.10) as references; the coupling constants are in Hz. ESIMS data were recorded on a Bruker Esquire 3000plus spectrophotometer, and HRESIMS data were obtained using Bruker APEX III 7.0T and APEXII FT-ICR spectrophotometers, respectively. Column chromatography was performed with silica gel (200–300 mesh, Qingdao Haiyang Chemical Factory) and Sephadex LH-20 (Pharmacia Co.) columns. TLC was carried out using silica gel GF254 (Qingdao Haiyang Chemical Factory) plates. HPLC was performed using an Agilent Chromatography C18 column (5 μm, 9.4 × 250 mm) with UV detection at 254 nm.

### 3.2. Fungal Material and Taxonomic Identification

**Figure 4:**
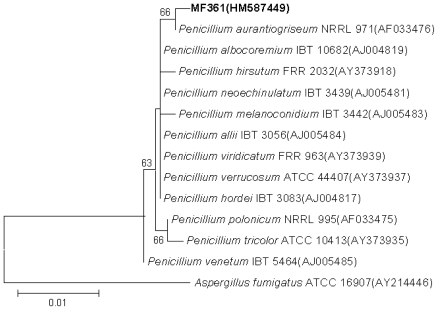
Neighbour-joining phylogenetic tree of strain MF361. Numbers at nodes indicate levels of bootstrap support (%) based on a neighbour-joining analysis of 1000 resampled datasets; only values >50% are given. NCBI accession numbers are given in parentheses. Bar, 0.01 nucleotide substitutions per site.

The marine derived fungus *Penicillium aurantiogriseum* was isolated from marine mud obtained in the Bohai Sea, and identified by morphology and sequence analysis of its internal transcribed spacer (ITS) region and 5.8S rDNA (GenBank accession number HM587449) using conventional primer pair ITS1(5″-TCCGTAGGTGAACCTGCGG-3″) and ITS4(5″-TCCTCCGCTTATTGATA-TGC-3″). The total genomic DNA of marine-derived fungus MF361 was extracted using the EZNA kit (Omega). The polymerase chain reaction product is 534 bp. The purified PCR products were sequenced (HuaDa Bio., Beijing, China). Multiple alignments with sequences of most closely related fungi and calculations of levels of sequence similarity were carried out using CLUSTAL W [29]. The phylogenetic tree was constructed using the neighbour-joining method [30], in MEGA 4.0 [31], as shown in Figure 4. The topology of the phylogenetic tree was evaluated by the bootstrap resampling method with 1000 replicates [32]. The fungus has been assigned the accession number MF361 in the culture collection at the Institute of Microbiology, Chinese Academy of Sciences, Beijing.

### 3.3. Large Scale Fermentation

The culture medium of the strain consisted of 30 g soybean powder and 120 g rice soaked in artificial seawater in a 500 mL erlenmeyer flask. Altogether, 14 flasks were cultured without shaking at 25 °C for 20 days before use.

### 3.4. Extraction and Isolation

The fermentation product was exhaustively extracted with EtOAc-MeOH-AcOH (80:15:5) to yield an extract. The crude extract was partitioned between EtOAc and H_2_O. The EtOAc layer (5.3 g) was applied to a column of silica gel using a stepped gradient solvent system of 0 to 100% MeOH/CH_2_Cl_2_ to afford 13 fractions. Fraction B (350 mg) was subjected to a Sephadex LH-20 column, eluted with petroleum ether-CH_2_Cl_2_-MeOH (5:5:1), to give five subfractions. Compounds **3** (8 mg, 0.15% yield) and **4** (12 mg, 0.23% yield) were obtained by separation of the subfraction B1 (62 mg) on a reversed-phase HPLC (Agilent Zorbax SB-C18 column, 5 μm, 9.4 × 250 mm) eluting with 80% CH_3_OH in water. While subfraction B3 (91 mg) was further purified on a RP-HPLC eluted with 65% MeOH in water to afford compounds **2** (17 mg, 0.32% yield), and a mixture of **5** and **6** (15 mg, 0.28% yield). Fraction D was also chromatographed on a Sephadex LH-20 column and eluted with the same solvent system as stated above, to afford five subfractions. Subfraction D1 (20 mg) was subjected to successive reversed-phase HPLC eluted with 50% acetonitrile in water, and further purified using 40% CH_3_OH in water to afford compound **1** (4 mg, 0.08% yield).

Verrucosidinol (**1**): yellow oil, stable in MeOH; [α]_D_^20^ −10.0° (c = 0.10, MeOH); UV (MeOH) λ_max_ (log ɛ) 231 (3.91), 288 (3.46) nm; IR *v*_max_ 2977, 2916, 2850, 1714, 1558, 1452, 1378, 1089, 1045, 1018 cm^−1; 1^H and ^13^C NMR data, see Table 1; ESIMS *m/z* [M + Na]^+^ 457; HRESIMS *m/z* 457.2195 [M + Na]^+^ (calcd for C_24_H_34_O_7_, 457.2197).

Verrucosidinol acetate (**2**): yellow oil, stable in MeOH; [α]_D_^20^ −34.0° (c = 0.10, MeOH); UV (MeOH) λ_max_ (log ɛ) 234 (3.84), 291 (3.45) nm; IR *v*_max_ 2973, 2935, 2877, 1745, 1667, 1597, 1452, 1372, 1230, 1138, 1087, 1043 cm^−1; 1^H and ^13^C NMR data, see Table 1; ESIMS *m/z* [M + Na]^+^ 499; HRESIMS *m/z* 499.2300 [M + Na]^+^ (calcd for C_26_H_36_O_8_, 499.2302).

### 3.5. Antimicrobial Assay

Fresh Mueller-Hinton broth medium (40 μL) was added to each well of a sterilized 96-well microtiter plate. To the test wells, 2 μL of the samples to be tested was added followed by 40 μL of the test strain solutions. The plate was incubated at 37 °C overnight. Anti-MRSA and PA activity of samples was checked by measuring and comparing the optical diversities of the blank control and test wells. All experiments were carried out in triplicate.

### 3.6. Antifungal and Synergistic Antifungal Assay

*Candida albicans* SC5314 was used as a test strain for antifungal and synergistic antifungal bioassay [33]. All experiments were carried out in flat bottom, 96-well microtiter plates (Greiner, Germany), using a broth microdilution protocol modified from the Clinical and Laboratory Standards Institute M-27A methods [34]. Overnight cultures were picked to prepare the strain solution with medium RPMI 1640 at the concentration of 1 × 10^4^ cfu/mL. To the test wells in 96 well plates, 2 μL of the samples to be tested was added followed by an additional 80 μL of the strain solution. The test plates were incubated at 35 °C for 16 hours. The antifungal positive control was ketoconazole and antifungal MICs were determined by measuring and comparing the optical densities of the blank control and test wells. For the synergistic antifungal assay, a quarter of the normal antifungal MIC of ketoconazole was supplemented into the strain solution, and the other procedures were the same as the antifungal assay. All experiments were carried out in triplicate.

## 4. Conclusions

Since the initial discovery of penicillin, many new bioactive compounds have been identified from the genus *Penicillium* [12,16,17]. As drug discovery expands into previously underexplored territories, such as the marine environment, more new compounds will be obtained, for example, the three quinazoline alkaloids (aurantiomides A–C), isolated from sponge-derived *P. aurantiogriseum* [16]. The secondary metabolites verrucosidinol (**1**) and verrucosidinol acetate (**2**) reported in this study are new members of the pyrone-type polyketides. Although they did not show significant bioactivity in three of the antimicrobial assays, in consideration of their structural similarity to the mycotoxin verrucosidin, they could potentially have other unexplored bioactivities.

## Figures and Tables

**Table 1:** NMR (500 MHz, CDCl_3_) Spectroscopic Data of verrucosidinol (**1**) and verrucosidinol acetate (**2**).

	verrucosidinol (1)				verrucosidinol acetate (2)			

Position	*δ*_C_	*δ*_H_^a^ (*J* in Hz)	HMBC^a^	NOESY	*δ*_C_	*δ*_H_^a^ (*J* in Hz)	HMBC^a^	NOESY
1	165.0, qC				164.3, qC			
2	110.4, qC				110.9, qC			
3	169.2, qC				168.5, qC			
4	111.8, qC				111.5, qC			
5	159.7, qC				157.5, qC			
6	78.8, qC				78.2, qC			
7	79.8, CH	4.61, s	5, 6, 8, 9, 18, 19	9, 17	82.1, CH	5.43, s	6, 8, 9, 19, 26	9, 17
8	133.9, qC				130.4, qC			
9	134.3, CH	5.87, s	7, 11, 19, 20	7	135.4, CH	5.85, s	7, 11, 19, 20	7
10	134.4, qC				134.3, qC			
11	133.0, CH	5.43, s	9, 12, 13, 20, 21,	13, 23	133.2, CH	5.41, s	9, 12, 13, 20	13, 23
12	80.1, qC				80.0, qC			
13	67.5, CH	3.43, s	12	11, 22	67.4, CH	3.41, s	12	11, 22
14	67.4, qC				67.4, qC			
15	76.7, CH	4.12, q (7.0)	12, 13	21	76.7, CH	4.11, q (7.0)	12, 13	21
16	10.2, CH_3_	2.01, s	1, 2, 3		10.2, CH_3_	2.02, s	1, 2, 3	
17	9.9, CH_3_	2.21, s	3, 4, 5	7	10.1, CH_3_	2.20, s	3, 4, 5	7, 25
18	23.4, CH_3_	1.40, s	5, 6, 7	19	23.8, CH_3_	1.55, s	5, 6, 7	19
19	14.8, CH_3_	1.82, s	7, 8, 9,	18	15.4, CH_3_	1.80, s	7, 8, 9	18
20	18.6, CH_3_	1.89, s	9,10,11		18.4, CH_3_	1.87, s	9, 10, 11	
21	21.9, CH_3_	1.41, s	11, 12, 13	15	21.8, CH_3_	1.39, s	11, 12, 13	15
22	13.8, CH_3_	1.47, s	13, 14, 15	13, 23	13.8, CH_3_	1.46, s	13, 14, 15	13, 23
23	18.8, CH_3_	1.18, d (7.0)	14, 15	11, 22	18.8, CH_3_	1.16, d (7.0)	14, 15	11, 22
24	60.3, CH_3_	3.79, s	3		60.3, CH_3_	3.78, s	3	
25					21.0, CH_3_	2.06, s	26	17
26					169.4, qC			

a HMBC NMR correlations (optimized for 8 Hz) are from proton(s) stated to the indicated carbon.
